# From morphological heterogeneity at alveolar level to the overall mechanical lung behavior: an in vivo microscopic imaging study

**DOI:** 10.1002/phy2.221

**Published:** 2014-02-07

**Authors:** Enrico Mazzuca, Caterina Salito, Ilaria Rivolta, Andrea Aliverti, Giuseppe Miserocchi

**Affiliations:** 1 TBM Lab, Dipartimento di Elettronica, Informazione e Bioingegneria, Politecnico di Milano, Milano, Italy; 2 Department of Health Sciences, Università di Milano‐Bicocca, Monza, Italy

**Keywords:** Alveolar mechanics, in vivo microscopy, multiscale

## Abstract

In six male anesthetized, tracheotomized, and mechanically ventilated rabbits, we imaged subpleural alveoli under microscopic view (60×) through a “pleural window” obtained by stripping the endothoracic fascia and leaving the parietal pleura intact. Three different imaging scale levels were identified for the analysis on increasing stepwise local distending pressure (*P*_ld_) up to 16.5 cmH_2_O: alveoli, alveolar cluster, and whole image field. Alveolar profiles were manually traced, clusters of alveoli of similar size were identified through a contiguity‐constrained hierarchical agglomerative clustering analysis and alveolar surface density (ASD) was estimated as the percentage of air on the whole image field. Alveolar area distributions were remarkably right‐skewed and showed an increase in median value with a large topology‐independent heterogeneity on increasing *P*_ld_. Modeling of alveolar area distributions on increasing *P*_ld_ led to hypothesize that absolute alveolar compliance (change in surface area over change in *P*_ld_) increases fairly linearly with increasing initial alveolar size, the corollary of this assumption being a constant specific compliance. Clusters were reciprocally interweaved due to their highly variable complex shapes. ASD was found to increase with a small coefficient of variation (CV <25%) with increasing *P*_ld_. The CV of lung volume at each transpulmonary pressure was further decreased (about 6%). The results of the study suggest that the considerable heterogeneity of alveolar size and of the corresponding alveolar mechanical behavior are homogenously distributed, resulting in a substantially homogenous mechanical behavior of lung units and whole organ.

## Abstract (precis)

Alveolar mechanics of rabbit lungs was studied by using a methodological approach, which strictly preserves the in vivo physiological setting, leaving the pleural cavity intact. Data from direct in vivo microscopic imaging of alveoli at different distending pressures were analyzed by novel geostatistical analyses to evaluate the topological dependence of heterogeneity in alveolar size, shape, and compliance. Results suggest that the mechanical behavior found by scaling up from single alveoli, to alveolar clusters and to the whole organ results from a nontopological‐dependent morphological and mechanical heterogeneity at microscale level.

## Introduction

Lung parenchyma is considered as a system composed by polyhedral alveoli opening on the lumen of alveolar ducts (Wilson and Bachofen 1982). The tissue structure surrounding alveoli is a complex interconnected network of macromolecules with viscoelastic and elastic properties. Alveoli also present a liquid interface with air implying the existence of surface forces. Essentially, whole mechanical lung behavior reflects the mechanical properties of single alveoli whose distending pressure is given by the sum of an elastic component, developed by the parenchymal structures, and a pressure component reflecting surface forces. Several studies have considered how considerable perturbation of physiological condition at lung periphery affect the intrinsic viscoelastic properties and/or surface forces creating regional heterogeneities in the mechanical behavior. Conditions studied include remarkable decrease in airways caliper following bronchoconstriction (Tgavalekos et al. 2005), chemical destruction of the elastic component (Suki et al. 2003), and acute lung injury (Allen et al. 2005). A recent article by Wilson (2013) proposed that, in physiological conditions, regional differences in volume oscillations may actually result from the complex interaction between the stiffness of the elastic elements of the alveoli and surface tension relaxation. We wished to provide a contribution to this hypothesis, addressing specifically two related questions: (1) assess differences in alveolar absolute and specific compliance: the former is defined as the change in volume over change in distending pressure, while the latter is defined as the absolute compliance over initial alveolar volume and represents the intrinsic mechanical property of the elastic components; (2) how the potential heterogeneity in alveolar mechanical properties impacts on whole lung mechanics. To this aim, we developed an experimental method that allowed us to image subpleural airway terminal units keeping pleural space intact; this approach allowed a clear and neat view of unrestrained subpleural structures exposed to changing physiological local distending pressure. We estimated alveolar compliance under static conditions, thus neglecting a phase shift between elastic and surface forces, as hypothesized by Wilson (2013).

## Materials and Methods

### Animal preparation

A general consensus for the experimental procedures used in our research activity was obtained from Milano‐Bicocca University Ethical Committee. Experiments were performed on six adult (New Zealand White) male rabbits (weight range 1–1.5 kg) anesthetized with a bolus of 2.5 mL/kg of a saline solution containing 0.25 g/mL of urethane plus 10 mg/mL of pentobarbital sodium injected into an ear vein. The absence of an eyelid closure reflex was used as an estimate of the proper level of anesthesia during the experiment. When necessary, subsequent doses of 0.5 mL of anesthetic were administered through a venous line. Tracheostomy was performed, and animals were intubated. Before connecting the rabbit to the ventilator, paralysis was accomplished by pancuronium bromide (1 mg/kg body weight initial dose, supplemented by 0.33 mg/kg every 40 min). Mechanical ventilation was provided with a tidal volume (VT) of ~20 mL and a frequency of 16 breaths/min. The inspiratory flow signal was measured by a Fleisch‐type pneumotachograph (Sensym, Milpitas, CA) and integrated in order to obtain lung volume; volume drift was avoided by correcting flow offset. Tracheal and esophageal (through esophageal balloon) pressures were measured by pressure transducers. Lung volume values were normalized to their maximum value, assumed at 20 cmH_2_O.

**Figure 1.**
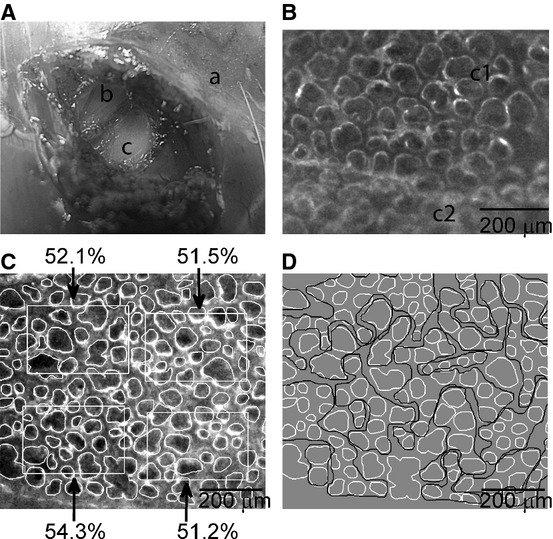
Experimental preparation. (A) Macrophotography on creating a “pleural window”: (a) intact chest wall, (b) intercostal muscle layer, (c) denuded portion of parietal pleura (“pleural window”). (B) Part of a pleural window under the microscopic view with a magnification of 60×: alveoli are clearly visible where the endothoracic fascia was stripped off the denuded parietal pleura (c1), as compared to the unstripped portion (c2). (C) An example of manually segmented alveolar units (digital zoom of a 60× magnified microscopic image) at *P*_ld_ = 4.5 cmH_2_O. Four nonoverlapped ROI are shown together with the corresponding percentage values of alveolar surface (alveolar surface density, ASD). (D) The same alveolar population of (C) with manually traced alveolar clusters (black lines) obtained by the contiguity‐constrained hierarchical clustering analysis. Alveoli not belonging to any clusters are not encircled.

An “intact pleural window” was prepared in the sixth intercostal space to allow a view of the lower lobe where the cardiac artifacts are relatively small. The skin and superficial muscles on the right side of the chest were resected and, when reaching the layer of the intercostal muscles (indicated by “b” in Fig. 1A), a surface area of about 0.5 cm^2^ was freed from muscles down to the endothoracic fascia. Under stereomicroscopic view and with fine forceps, the endothoracic fascia was then carefully stripped, exposing the transparent parietal pleura (about 10 *μ*m thick) through which freely moving subpleural alveolar structures could be neatly visualized (area indicated by “c” in Fig. 1A). The microscopic image in Figure 1B highlights the difference between the stripped area (indicated by “c1”) and the unstripped one (indicated by “c2”).

### Experimental protocol

#### Image acquisition

The animal was placed in left lateral decubitus under the field of a fixed microscope (Nikon's Eclipse FN1, Nikon Corporation Instruments Company, Tokyo, Japan) connected to a video camera (CoolSNAP_*EZ*_, Photometrics, Tucson, AZ) which was interfaced through a IEEE‐1394 data‐transfer interface card with a personal computer equipped with an image‐processing software (MetaMorph^®^ System, Molecular Devices, Sunnyvale, CA). Total microscopic magnification was 60×. A LED ring‐light illuminator was anchored to microscopic optics to provide a uniform lighting of the alveolar field. The depth of field of the objective used (4×, NA = 0.1) was about 50 microns. As we focused the visceral pleura, the microscopic image referred to a depth of 50 microns within the lung parenchyma. We also refer to common histology (hematoxilin–eosin) performed in our laboratory on sagittal sections of the lung fixed at a transpulmonary pressure corresponding to functional residual capacity (FRC) to estimate the average thickness of the visceral pleura (Rivolta et al. 2011).

We imaged the alveolar units of the right lower lobe, where no interlobar fissures crossed the optical field. Alveolar units included both alveoli and alveolar sacs. The change in alveolar size was assessed during two/three recruitment–derecruitment maneuvers: mechanical ventilation was stopped at the end of expiration and lung was recruited from FRC up to an alveolar pressure (*P*_alv_) of 20 cmH_2_O (100% of inspiratory capacity, IC) by injecting air through a syringe and controlling intratracheal pressure. Each step involved an increase/decrease of 4 cmH_2_O. During the maneuver, an image was acquired at each pressure step, when quasi‐static conditions were reached (about 2 sec for each pressure level). The displacement of the alveolar field under microscopic view required a manual focus adjustment at each pressure step. At the end of the experiment, the animals were euthanized with an anesthetic overdose.

#### Morphological and morphometric analysis of alveolar units

For each image, alveolar units (both alveoli and alveolar sacs) were manually segmented along the sharp gradient in optical density between the gas phase and the interstitial tissue in order to derive individual alveolar surface area (Fig. 1C) and to obtain then their frequency distributions. The latters did not include alveolar sacs, whose geometry depart from circularity. Given a rectangular region of interest (ROI) on the image (with a variable size around 200 × 300 micron, Fig. 1C), the alveolar surface density (ASD) was estimated as the ratio of alveolar surface area to the area of the ROI; up to four equally sized, minimally overlapping, randomly selected ROIs containing at least 10 alveoli were analyzed for each image to obtain an average value for ASD at each alveolar pressure.

#### Local distending pressure

Given the animal position, the imaged lung units of the right lower lobe were placed in the less‐dependent portion of the pleural cavity, that is, at 100% of lung height. For these units, it was possible to obtain an estimate of the local distending pressure (*P*_ld_) defined as: 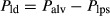
where *P*_lps_ is the local pleural pressure. *P*_lps_ was derived from figure 7 of Agostoni and Miserocchi (1970), a study that reported direct measurements performed at different lung heights and for different *P*_alv_ values in the same species.

#### Cluster and fractal analysis

Cluster analysis was carried out to describe the shape and topological distribution of clusters of alveoli having similar area values and was based on contiguity‐constrained hierarchical agglomerative clustering (Recchia 2010). This analysis allows to group alveoli based on two inclusion principles: the first one is based on similarity in surface area, the second is contiguity.

The contiguity constraint is initially computed on single alveoli that are considered adjacent if their centroids are not farthest than two times their average surface diameter and no other alveoli are found on the line joining their centroids. A representative example of this clustering analysis is presented in Figure 1D, where alveolar clusters are manually encircled by black lines. Clusters were geometrically characterized by computing their circularity, defined as 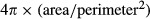 grouping data at *P*_0_.

### Statistical analysis

Kolmogorov–Smirnov normality test was performed on alveolar populations using Matlab^®^. Spatial statistical analysis (semivariogram) of areas of single alveoli was performed to express the relationship of the respective variances as a function of increasing distance (“lag”) between individual alveoli, in order to assess whether there is a distance dependence for alveolar area distribution. Logarithms of area values were used to obtain normal distributions, on which semivariogram analysis was carried out, using geoR package (R, version 3.0.1). Semivariogram could be computed only up to a maximum distance corresponding to half the window size (about 500 *μ*m).

## Results

Data will be presented in accord to three scale levels: alveoli, cluster of alveoli, and overall image/whole lung.

### Alveolar level

**Figure 2.**
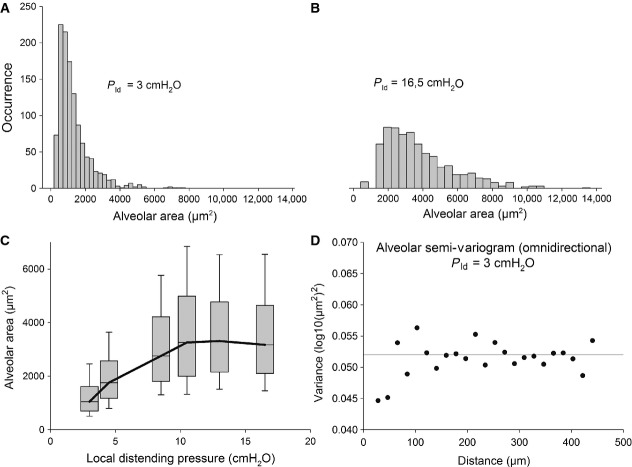
(A and B) Distributions of alveolar areas at *P*_ld_ = 3 and 16.5 cmH_2_O (*P*_0_ and *P*_1_, respectively), obtained by pooling data from all rabbits. (C) Box plot describing the alveolar surface areas from all rabbits at different levels of alveolar pressure (only the inflation limb is shown). For each pressure, the gray box shows a 25–75% percentile range; the median value is represented by the horizontal bar. The whiskers above and below the gray boxes encompass 80% of the variability in area values. (D) Semivariogram of alveolar areas at *P*_0_.

Representative distributions of alveolar areas pooled from all rabbits are presented in Figure 2A and B for *P*_alv_ = 0 cmH_2_O and *P*_alv_ = 20 cmH_2_O, corresponding to local distending pressure *P*_ld_ of 3 and 16.5 cmH_2_O, respectively referred, from this point onwards, as *P*_0_ and *P*_1_. The total number of alveoli analyzed for each image ranged between 100 and 150, providing an overall number of alveoli in a range of 1000 at each pressure. These distributions were found to be right‐skewed, that is smaller alveoli are more numerous than the greater ones. The semivariogram presented in Figure 2C shows that the variance of the alveolar areas at *P*_0_ is independent of topological distribution, as it oscillates by about ±3% around the variance of the alveolar population, named “sill.” Similar results were obtained for the same analysis carried out at increased distending pressure, with an average oscillation of ±7% around the “sill.”

Figure 2D shows that the median values of alveolar areas, pooling data from all rabbits, increase with *P*_ld_, with a remarkably increasing wide variability (expressed by the 25th–75th percentiles, dashed areas). Kolmogorov–Smirnov normality tests failed for distributions at all distending pressures. Also log‐normality test, performed by applying normality test on the logarithm of data, failed for each distribution. The coefficient of variation (CV) of each distribution was computed by dividing the interquartile range (75th percentile value minus 25th percentile value) by the median value and did not show any specific trend to increase with increasing distending pressure: for local distending pressure of 3, 4.5, 8.5, 10.5, 13, and 16.5 cmH_2_O on the inflation limb, the CV values were 88%, 79%, 87%, 92%, 79%, 80%, respectively.

### Cluster level

**Figure 3.**
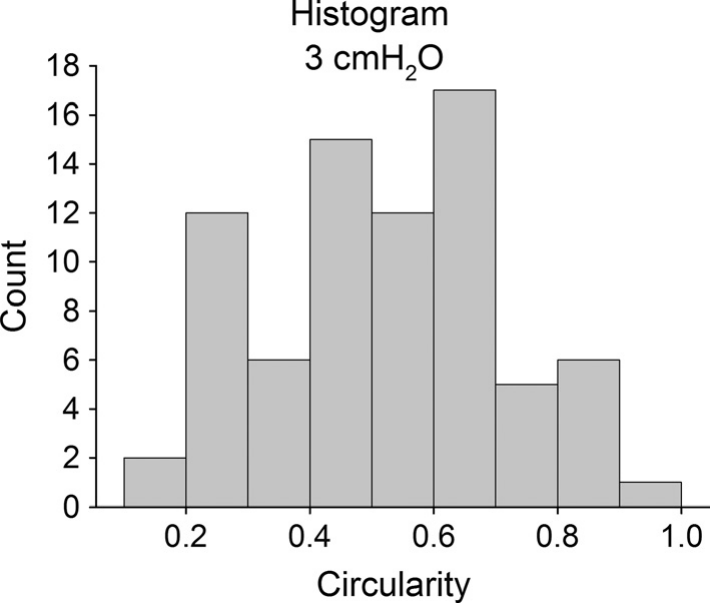
Histogram of circularity values for alveolar clusters at *P*_0_.

Figure 1D allows to appreciate the complex matching of clusters due to their highly variable shape. Figure 3 shows that cluster shapes are far from circularity and quite dispersed.

### Whole image level

**Figure 4.**
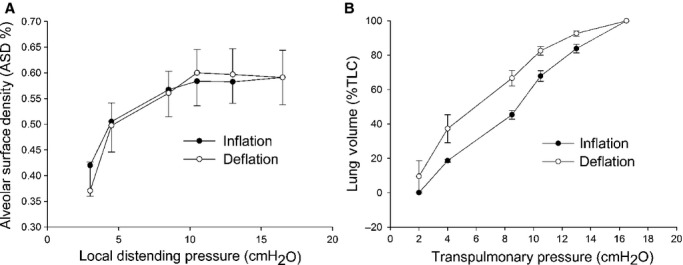
(A) Alveolar surface density (ASD) as a function of the local distending pressure. Mean values of data pooled from all rabbits are presented with ±SD. (B) Transpulmonary pressure versus lung volume curve (normalized to TLC), obtained as the average of individual rabbit pressure–volume curves.

The average values of ASD ± SD, obtained by pooling values from all animals, is reported in Figure 4A and shows an increase up to a *P*_ld_ of 10 cmH_2_O, with CV at each distending pressure not exceeding 25%. Figure 4B shows the overall lung volume versus distending pressure curve and allows to appreciate that the average CV of volume is further reduced to about 6%.

## Discussion

Previous attempts of imaging of subpleural alveoli to some extent largely interfered with unrestrained alveolar movement (Liu et al. 2011). For example, in Schwenninger et al. (2008) and Tabuchi et al. (2008), the visceral pleura was stuck to a transparent window. By using a tomographic technique as optical coherence tomography (Meissner et al. 2010) or confocal microscopy (Namati et al. 2008), a three‐dimensional (3D) analysis could be performed. In this case, however, less pressure steps could be done in in vivo experiments due to the time required by the 3D acquisitions.

The experimental approach used in this study allowed to investigate the morphology of the subpleural alveoli by maintaining pleural space mechanics intact. A notable limitation was that it was not possible to follow the morphological change in the same units. On the other side, the method had the advantage to allow unrestrained movement of subpleural units, exposed to physiological distending pressure, by preserving the integrity of the respiratory system. This allowed to relate the morphology of the alveoli, assessed through a two‐dimensional analysis, to the expected local distending pressure (Agostoni and Miserocchi 1970). This last point is of remarkable importance as it is not available by other techniques used to investigate the morphology of individual alveoli within the lung parenchyma. Another potential limitation is represented by the fact that only subpleural units were studied and the discussion concerning their mechanical behavior does not allow us to extrapolate to the rest of the parenchyma. In fact, the areas of the subpleural alveoli are controlled by the lines of attachment of alveolar walls to the pleura and the displacements of these attachments relate to the displacement of the pleural membrane which has been shown to expand isotropically as lung volume increases (Lehr et al. 1985). However, recalling data from Gil et al. (1979), no differences in surface area expansion have been described between subpleural and core alveoli from fixed rabbit lungs. Finally, our imaging system did not allow a 3D reconstruction. Given the depth of field of our objective (50 microns), we essentially imaged the terminal airways that may include both alveoli as well as alveolar sacs, as confirmed by sagittal histological sections from our lab; as specified in the methods, we only considered surface area values of the alveoli. One may wonder whether the observed heterogeneity could in part be due to the fact that the focal plane cuts the alveoli at different positions with respect to their centroids. This would lead to an error in the measured areas, representing an underestimate relative to the area corresponding to the plane of the alveolar centroid. From the histological sections, we also estimated an average thickness of the visceral pleura of 10 ± 2 microns, so that all alveolar walls are essentially at equal distance from the pleural surface. Therefore, the underestimate would only occur for larger alveoli when the centroid is much below the focal field. This bias, however, is presumably constant at all pressures, so that it would not affect relative changes in alveolar areas observed in increasing distending pressure.

**Figure 5.**
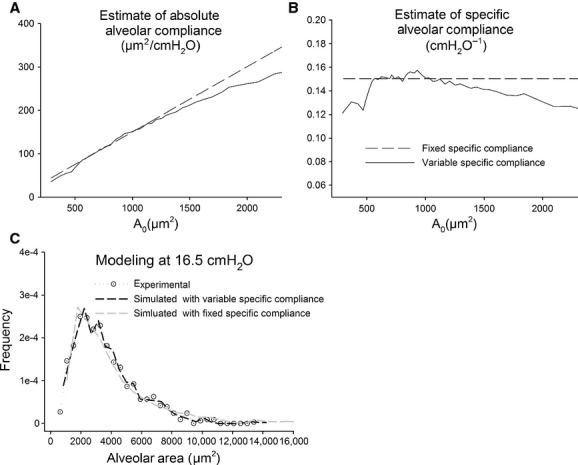
(A) Absolute compliance estimated by comparing subpopulations drawn from alveolar area distributions at *P*_0_ and *P*_1_ (continuous line). (B) Specific compliance derived from the absolute compliance, as detailed in the text (continuous line). Dashed line in (A) and (B) correspond to the assumption of a constant specific compliance. (C) Modeling of the experimental alveolar area distribution at *P*_1_ (white dotted points), assuming either a variable (black dashed line) or fixed (gray dashed line) specific compliance.

By considering the histograms of Figure 2A, we will attempt an interpretation of the change in distribution of the alveolar caliper with increasing *P*_ld_ to derive indications on the mechanical properties of the alveoli. In particular, we propose to interpret our data in terms of alveolar distensibility. The latter property was estimated by the ratio of the increase in alveolar surface area for an increase in alveolar distending pressure from *P*_0_ to *P*_1_, namely *C*_abs_ = (*A*_1_ – *A*_0_)/(*P*_1_ − *P*_0_), which represents an average index of alveolar absolute compliance in the range of pressure considered. We also defined specific alveolar compliance as *C*_sp_ = *C*_abs_/*A*_0_. As we cannot follow the same alveoli on increasing *P*_ld_, we attempted to derive both absolute and specific alveolar compliance from the frequency distribution of alveolar areas at *P*_0_ and *P*_1_, as detailed in the Appendix A1. We implicitly assume that, within a frequency distribution at a given *P*_ld_, alveoli of different size are actually all exposed to *P*_ld_, for mechanical equilibrium. Figure 5A shows that the computed estimate of *C*_abs_ (continuous line) increases sevenfold for a range of increase in *A*_0_ including 90% of alveolar population. Correspondingly, one can appreciate that specific compliance *C*_sp_ (Fig. 5B, continuous line) remains essentially steady (0.12 ÷ 0.15 cmH_2_O^−1^) for the same range of alveolar population. To simplify the model, we assumed a constant value for specific compliance (see Appendix A1), reported by the dashed lines in Figure 5B. Obviously, a constant value for *C*_sp_ implies a linear increase in *C*_abs_ (Fig. 5A, dashed line). To validate this assumption, we show in Figure 5C that a similar satisfactory modeling of the experimental distribution (white points) at *P*_1_ was found considering either the variable or fixed specific compliance (dashed black and gray line, respectively). One shall comment that the derived absolute compliance values obviously reflect the shape of the experimental area distribution curves and the modeling assumption of the correspondence of the *i*‐th subpopulations at various *P*_ld_.

### Consideration relative to the difference in the mechanical behavior of lung units

Results of this study reveal that the considerable heterogeneity in alveolar size (Fig. 2C) does not show a topological dependence, as demonstrated by the semivariogram of Figure 2D, at least over a limited range of distance (500 *μ*m). Furthermore, the clusters also appear to be interweaved (Fig. 1D), having a highly variable shape (Fig. 3). Therefore, we assume that alveoli are not independent entities but are exposed to mechanical interdependence regardless of the fact that alveoli are or are not subtended by the same airway.

Obviously, we are far from extending our considerations to the whole lung functions where inhomogeneous behavior is known to be present concerning various aspects such as gas mixing, regional volume change, due to several interacting factors, such as gravity, airway resistance, etc. Interestingly, scaling up from single alveoli to randomly selected alveolar regions, a relatively homogenous mechanical behavior with minimal hysteresis of overall alveolar expansion is observed, as suggested by the low CV of ASD (Fig. 4A). This occurs despite the fact that differences in alveolar morphology imply differences in absolute compliance (Fig. 5). Scaling up to the level of the whole lung, a relative homogenous behavior is also observed given the low CV of lung volumes at various transpulmonary pressures. Thus, to summarize, we measured the CV of volumes at alveolar, ROI and whole lung levels and found that heterogeneity is maximal at the alveolar level. Note that for the whole lung expansion, various structures with different mechanical properties become involved (alveolar ducts, bronchioles, bronchi). In fact, on comparing Figure 4A and B, one can deduce that above 10 cmH_2_O a contribution to increase in lung volume must come from lung structures other than the alveoli under analysis and this also implies an increase in hysteresis from ASD to whole organ, confirming previous findings obtained on isolated lung lobes using a synchronized stroboscopic photography (Lehr et al. 1985). In conclusion, to answer the questions addressed in the introduction, our data suggest that the mechanical behavior found by scaling up to lung units and to the whole organ results from a non topological‐dependent morphological and mechanical heterogeneity at microscale level (alveoli and cluster). The relative constancy of the estimated alveolar specific compliance could be an important factor to account for morphofunctional homogeneity on increasing scale level. Indeed, we might consider that, in an interdependent environment, a homogenous distribution of specific compliance of elastic components might reduce interregional differences in parenchymal forces during lung volume changes. Of course, this working hypothesis requires an experimental validation by following the expansion of the same alveoli on increasing distending pressure. Regarding the fact that no hysteresis was observed in alveolar areas, we can make the following comment: on one side, this may be due to the fact that we could actually not follow the same alveoli; on the other, we may suppose that hysteresis arises from other structures such as alveolar ducts. The hysteresis observed in lung volume change, despite a small CV of lung volume (Fig. 4B), may be ascribed to a multiscale effect of hysteretic factors on lung units having different mechanical properties. This can be the object of a further study requiring the use of imaging techniques allowing to explore internal parenchymal structures.

## Conflict of Interest

None declared.

## Appendix

### Appendix

We defined subpopulations from the frequency distributions at *P*_0_ and *P*_1_ by dividing the overall populations in 50‐quantiles and we assumed that each *i*‐th subpopulation at *P*_0_ fell in the corresponding *i*‐th subpopulation at *P*_1_. The absolute compliance of the *i*‐th subpopulation,

, from *P*_0_ to *P*_1_ was estimated from the increase in median value of the subpopulation itself (

), divided by the increase in *P*_ld_:

The specific compliance index was given by

A constant value of specific compliance was estimated by

(

where

and

are the median value of distributions at *P*_0_ and *P*_1_)
